# The Use of Viral Vectors for Gene Therapy and Vaccination in Tuberculosis

**DOI:** 10.3390/ph16101475

**Published:** 2023-10-16

**Authors:** Dulce Mata-Espinosa, Jacqueline V. Lara-Espinosa, Jorge Barrios-Payán, Rogelio Hernández-Pando

**Affiliations:** Sección de Patología Experimental, Instituto Nacional de Ciencias Médicas y Nutrición Salvador Zubirán, Vasco de Quiroga 15, Belisario Domínguez Sección 16, Tlalpan, Mexico City 14080, Mexico; jvle_29031991@comunidad.unam.mx (J.V.L.-E.); jorge.barriosp@incmnsz.mx (J.B.-P.)

**Keywords:** tuberculosis, gene therapy, adenovirus, vaccines

## Abstract

Tuberculosis (TB), an infection caused by *Mycobacterium tuberculosis (Mtb)*, is one of the primary causes of death globally. The treatment of TB is long and based on several drugs, producing problems in compliance and toxicity, increasing *Mtb* resistance to first-line antibiotics that result in multidrug-resistant TB and extensively drug-resistant TB. Thus, the need for new anti-TB treatments has increased. Here, we review some model strategies to study gene therapy based on the administration of a recombinant adenovirus that encodes diverse cytokines, such as IFNγ, IL12, GM/CSF, OPN, TNFα, and antimicrobial peptides to enhance the protective immune response against *Mtb*. These models include a model of progressive pulmonary TB, a model of chronic infection similar to latent TB, and a murine model of pulmonary *Mtb* transmission to close contacts. We also review new vaccines that deliver *Mtb* antigens via particle- or virus-based vectors and trigger protective immune responses. The results obtained in this type of research suggest that this is an alternative therapy that has the potential to treat active TB as an adjuvant to conventional antibiotics and a promising preventive treatment for latent TB reactivation and *Mtb* transmission. Moreover, Ad vector vaccines are adequate for preventing infectious diseases, including TB.

## 1. Introduction

Along with malaria and HIV/AIDS, tuberculosis (TB) is one of the most deadly diseases in the world and has had an immense socioeconomic impact on humanity [1]. TB is spread through the air and is brought on by a single agent called *Mycobacterium tuberculosis* (*Mtb*) [2]. Although the bacteria typically affects the lungs, it can also harm any organ, including the kidney, spine, and brain [3]. The causative agent *Mtb* is a facultative intracellular pathogen [2] and fewer than 10% of people who have been exposed to *Mtb* develop primary TB infection within two years. On the other hand, latent infection, which develops without exhibiting the clinical signs or symptoms of the disease, is more common in exposed persons [4]. The initial or primary infection is established in the lungs during childhood, and, in most cases, it is controlled by the immune system [5]. In this primary TB infection, even in cases handled by the immune system, not all the bacteria are eliminated; some bacilli remain in the tissues in a quiescent state with little or no reproductive activity for the rest of the infected individual’s life. This infectious state is called latent infection and is clinically asymptomatic [5].

Globally, TB results in 10 million new cases of active TB each year and 1.5 million fatalities, and a quarter of humanity has a latent infection, which makes it the most important infectious disease caused by a single bacterial agent in the world [6]. Since more than a century ago, Bacille Calmette–Guérin (BCG) has been the only TB vaccination that is widely accessible. Infants receive intradermally the BCG vaccine, a live, attenuated version of *M. bovis* [7]. Although the BCG vaccine is a preventive measure, protection against TB is frequently lost by adolescence or early adulthood, and it is ineffective in preventing pulmonary TB. Due to this, TB infection is spread or reactivated. Numerous investigations on the BCG vaccine have revealed that BCG is unable to induce the development of a variety of different, diverse, and cell-mediated immune responses that would be able to eliminate *Mtb* infection [7]. Therefore, there is a pressing need to create novel vaccines that will cause trained and heterologous, long-lasting immune responses and provide protection against *Mtb* infection.

TB is treated with a combination of 4 antibiotics for 6 to 9 months, resulting in a high treatment-abandonment rate. This situation has promoted relapses and the emergence of Multidrug-resistant TB (MDR-TB), extensively drug-resistant TB (XDR-TB), and total drug-resistant TB (TDR-TB) [8,9]. MDR-TB is a TB disease in which the *Mtb* strain resists two of the most often used drugs, isoniazid and rifampicin [8,9]. The term “XDR-TB” refers to MDR-TB that has added bacillary resistance to any fluoroquinolone (FQ) and at least one of the three second-line injectable medications (i.e., amikacin (AMK), kanamycin (KAN), and capreomycin (CAP) [9]. TDR-TB is an XDR-TB that is resistant to both first- and second-line treatments that are typically used, as well as rifabutin (RFB), clofazimine (CLO), dapsone, clarithromycin (CLR), thiacetazone (THZ), bedaquiline (BDQ), and delamanid (DMD) [9]. MDR, XDR, and TDR-TB complicate treatment by increasing costs and generating more toxic effects and higher mortality [8,9]. A shorter and more efficient course of TB treatment may be achieved with host-directed therapy administered alone or in conjunction with other treatments. Host-directed therapies offer an alternate strategy by interfering with host cellular processes necessary for pathogen survival or reproduction by targeting the host immune response to infection (immunotherapy), which can either suppress the harmful immune response or, more commonly, enhance the protective immune response [10].

Gene therapy is a host-directed therapy that mediates the effects by transcription and/or translation of transferred genetic material by administering nucleic acids, viruses, or genetically engineered microorganisms [11]. Recombinant adenovirus (Ad) vectors are compelling gene delivery systems. Ad vectors provide several benefits, including their straightforward manufacture at high titers, exceptional genome stability, low levels of vector genome integration, and well-studied virus biology. They can also efficiently transduce both dividing and non-dividing cells [12]. Therefore, the Ad gene transfer vectors are a promising strategy to increase the host immune response to infection during TB.

Although in vitro research is still relevant, in vivo models have offered significant insights because of the complexity of a living system. In this review, we aim to highlight various forms of adenoviral therapy that are based on experimental findings primarily from murine models of pulmonary TB, which may have a potential for later evaluation and use for preventing the reactivation of latent infections in people or to reduce chemotherapy in patients with active TB. We also discuss novel vaccines that use virus or particle-based vectors to deliver *Mtb* antigens and generate protective immune responses.

## 2. The Immune Response in Pulmonary TB

Innate and acquired immunity contribute to the control and elimination of *Mtb* [13]. When mycobacteria enter a host’s body through the airway, the host’s immune system recognizes them and can produce one of three effects. Complement factors may bind to mycobacteria and create a pore that results in the lysis of the microorganisms. At the same time, cells like neutrophils, macrophages, and dendritic cells (DCs) attempt to control the infection by engulfing the mycobacteria, which results in antigen presentation [14]. The bronchial epithelium also plays a significant role since it produces antimicrobial peptides that contribute to the elimination of bacteria [15,16]. As a result, an adaptive immune response is triggered, during which B-lymphocytes (BL) release cytokines, and *Mtb*-specific antibodies (Ab) with various effector functions target the bacteria. Such Ab synthesis is frequently carried out by T CD4^+^ lymphocytes, which change BL into plasma cells that produce Ab. While cytotoxic T CD8^+^ cells directly kill cells containing the TB bacillus, T CD4^+^ cells also aid in the intracellular elimination of mycobacteria in infected cells. These cellular elements that participate in immune protection against *Mtb* can be stimulated to induce more significant bacteria elimination and thus become immunotherapy targets [10,15]. The manipulation of cells like macrophages by the mycobacterium, where it can create a niche and multiply, as well as the manipulation of alveolar epithelial cells and neutrophils, which results in necrosis, are some of the different evasion methods that *Mtb* has developed against a host’s immune response. Additionally, it avoids phagolysosome formation, antigen presentation and processing, and a lymphocyte-mediated immune response that eliminates *Mtb* [14].

## 3. The Experimental Models of Pulmonary TB

Our line of research aims to characterize the immunological mechanisms that contribute to protection, progression, and tissue damage in TB. Describing these immunological processes allows an immunotherapeutic intervention, based on promoting protective activity and/or suppressing the mechanisms that facilitate the progression of the disease. Therefore, the control and prevention of TB can be substantially improved [10,16,17]. To achieve this objective, our research group has developed different experimental models related to pulmonary TB. Humanized mouse models have also been developed to study TB. These models helped develop the present understanding of TB’s pathophysiology, underlying mechanisms, and therapy.

### 3.1. The Experimental Model of Progressive Pulmonary TB

**Figure 1:**
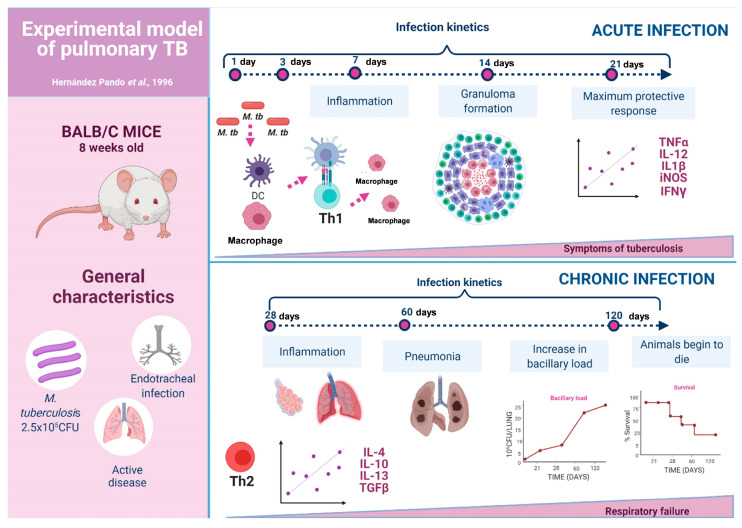
The experimental model of progressive pulmonary TB. In this model, BALB/C mice are infected via the endotracheal route with 2.5 × 10^5^ colony-forming units (CFU) of the H37Rv strain. Two stages are established through sacrifice kinetics on days 1, 3, 7, 14, 21, 28, 60, and 120 post-infection. The first corresponds to the protection stage (days 1–21), during which the bacillary load remains low, and there is no tissue damage (pulmonary pneumonia) or any mortality events. This stage is related to a high production of Th1-type cytokines, mainly IFNIγ, which permit bacterial control by activating various cell populations. In the second phase, or progressive (days 28–120), there is a high pulmonary bacillary load, extensive pneumonia, and high mouse mortality. This stage is related to the decrease in Th1 cytokines and the emergence of Th2-type cytokines and other immunosuppressive factors. These permit disease progression and culminate in animals’ death due to respiratory failure [18] (Created with BioRender.com (accessed on 10 July 2023).

The experimental model of progressive pulmonary TB is based on the use of genetically identical animals (Balb/c mice), which are infected by the natural route (intratracheal injection) with live and virulent bacteria (prototype strain H37Rv or MDR). This model is characterized by establishing two phases [18] (Figure 1). The first is the early phase, which corresponds to the first month of infection and is characterized by an inflammatory infiltrate of lymphocytes and macrophages in the alveolar–capillary interstitium, around venules and bronchi. During the second week post-infection, granulomas start forming, and one week later, they reach their maximum maturity [18,19]. These granulomas and the coexisting inflammatory infiltrate are mainly made up of Th-1 lymphocytes and activated TNFα and IL-1-producing macrophages, thereby maintaining low bacterial growth. Another fundamental component in controlling bacillary growth is antimicrobial peptides such as beta-defensins and cathelicidin, which are also highly produced during early infection [20].

The second stage of the disease in this experimental model is the advanced or progressive phase, characterized by a significant and progressive increase in the number of bacteria and areas of pneumonia with foci of necrosis and extensive fibrosis, which produce death [18,19]. From the immunological perspective, during this phase, the presence and activity of regulatory T cells and Th-2 lymphocytes increase significantly, and the production of their characteristic cytokines IL-10, IL-4, and IL-13 antagonize Th-1 cells, which favor the disease progression. In addition, macrophages have significant morpho-functional modifications; their cytoplasm is filled with lipid vacuoles and bacteria, and their production of TNFα and other protective factors decreases while increasing the production of anti-inflammatory and suppressive cytokines of cellular immunity, such as TGFβ and IL-10 [18,19]. During this advanced phase of the disease, there is also a pronounced decrease in the production of beta-defensins and cathelicidin [20].

Almost one-third of recorded cases of TB are extrapulmonary, including pleural TB, even though the pulmonary form of the disease is the most prevalent [21]. Both immunocompetent and immunocompromised persons can develop extrapulmonary TB [22]. The experimental model of progressive pulmonary TB could be used to establish a model of extrapulmonary TB. The extrapulmonary TB model could be created using an immunosuppressant. Giving corticosterone to mice before infection with *Mtb* could allow extrapulmonary TB to develop. Another alternative could be to use a TNFα blocker after infection with *Mtb*. We have demonstrated this using batimastat (BB-94), an inhibitor of the Tumour Necrosis Factor α Cleaving Enzyme (TACE) responsible for the processing of TNFα. The treatment for one month of BB-94 in the experimental model of pulmonary TB generated a mononuclear inflammatory infiltration in the pleura that spread to the pericardium and mediastinum, indicating the spread of mycobacteria [23]. The result of this experimental model would enable us to understand the pathophysiological mechanism of extrapulmonary TB and the possible treatments for this form of the disease.

### 3.2. Model of Chronic Infection Similar to Latent TB

**Figure 2:**
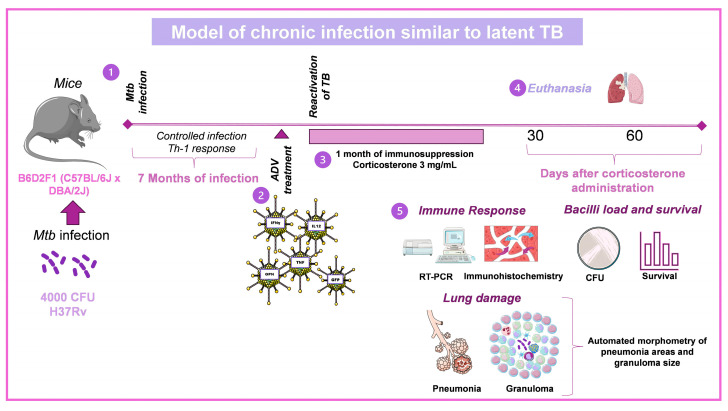
Model of chronic infection similar to latent TB and its reactivation prevention by recombinant adenovirus administration. (1) Groups of 8-week-old B6D2F1 female mice were infected via IT with 4000 CFU of *Mtb* strain H37Rv. (2) Once a latency state was established (7 months), a single dose of recombinant adenoviruses (Ad) via IT was administered to groups of mice. (3) After one 1-month post-treatment, latent TB reactivation was induced by administering corticosterone in drinking water for one month. (4–5) Lungs from control and Ad-treated mice were used to determine bacillary burdens by colony forming unit enumeration (CFU), cytokines production (RT-PCR, immunohistochemistry), and determination of tissue damage by automated histomorphometry [24]. Drawings from Servier Medical Art were used in some areas of the figure. Servier Medical Art by Servier is licensed beneath a CCA 3.0 Unported License (https://creativecommons.org/licenses/by/3.0/ (accessed on 7 May 2023).

Two other important aspects of TB are latent infection and transmission of *Mtb* to close contacts of the TB patient. In these two conditions, we have established two separate experimental models in which immunotherapeutic strategies can be designed and tested to avoid the reactivation of latent infection and prevent infection in healthy cohabitants. The similar latent infection condition model consists of intratracheally infecting F1 C57Bl/DBA hybrid mice with lower *Mtb* [24]. Animals develop granulomas with low and stable *Mtb* numbers, allowing the mice to grow and look physically healthy. Regarding the immune response, these animals show exclusive Th-1 activity and activated macrophages with high and constant TNFα production [24]. Suppose these mice are treated with 3 mg/mL corticosterone in drinking water. In that case, they are immunologically suppressed and, within a month, develop TB reactivation, characterized by a high increase in bacteria loads and progressive pneumonia [24] (Figure 2).

### 3.3. Murine Model of Mtb Transmissibility

**Figure 3:**
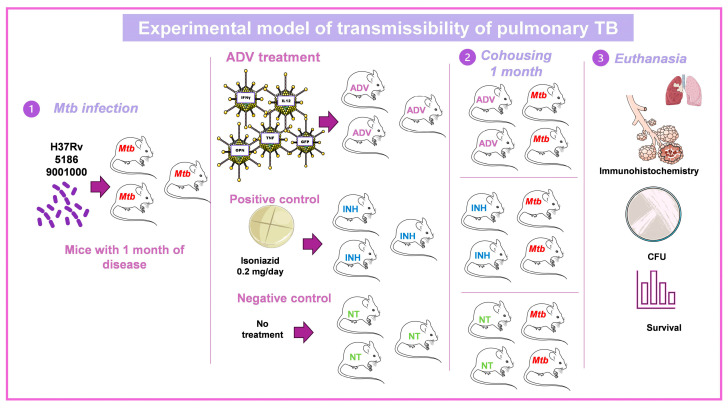
Experimental model of transmissibility of pulmonary TB and the efficiency of recombinant adenoviruses to prevent it. (1) Groups of 5 8-week-old male BALB/c mice are infected with a high dose of the H37Rv strain or a hypervirulent and highly transmissible strain *Mtb* (5186 or 9001000). A group of 5 mice is treated with a single dose of the adenoviruses, a treatment with isoniazid Monday, Wednesday, and Friday (positive control), or saline solution (negative control). (2) Both groups of mice are placed in the same box for one month. (3) The animals are euthanized, and the lungs are obtained to determine the bacillary load and the immunological response [25]. Drawings from Servier Medical Art were used in some areas of the figure. Servier Medical Art by Servier is licensed beneath a CCA 3.0 Unported License (https://creativecommons.org/licenses/by/3.0/ (accessed on 7 May 2023).

Our transmissibility model recreates the close-knit living situations experienced by TB patients and their household or work members [25]. This model consists of the cohousing in the same microisolator for one month of five infected mice with highly virulent and transmissible *Mtb* clinical isolates with five healthy animals. After one month, healthy animals become infected by close contact with the sick animals [25]. One dose of recombinant Ad is administrated by the IT route to healthy mice one day before cohousing with tuberculous mice, and after one month, Ad-treated mice are euthanized, and their lungs are used to determine CFU and tissue damage to determine the efficiency of gene therapy to prevent *Mtb* transmission [25] (Figure 3).

### 3.4. Humanized Mouse Models of TB

As previously described, several outstanding animal models for researching *Mtb* infection exist. However, these do not necessarily represent the pathology of TB in humans. The immunodeficient mice engrafted with healthy human cells and tissues (humanized mice) are tiny, preclinical animal models for studying human diseases [26]. Studies of cancer, regenerative medicine, graft-versus-host disease, allergies, immunology, and human infectious disease are all making considerable advances thanks to humanized mice [26]. Using humanized mice could result in the deployment of genuinely individualized healthcare in clinics.

**Figure 4:**
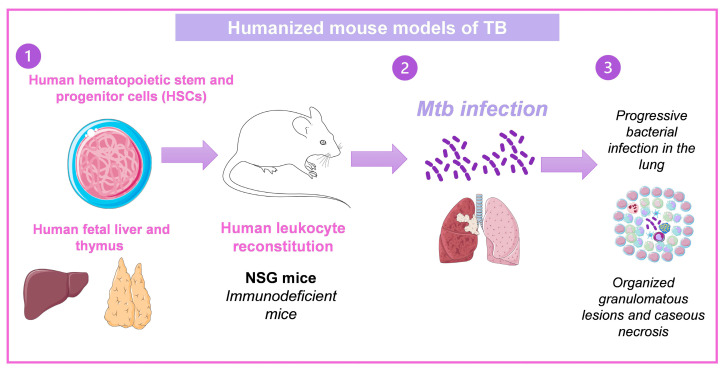
Humanized mouse models of TB. (1) In immunodeficient mice, human CD34+ HSCs derived from fetal liver are intravenously injected, or human fetal thymus and liver fragments are engrafted under the renal capsule of the kidney, and human CD34+ HSCs are intravenously injected from the autologous fetal liver. (2) After human leukocyte reconstitution in mice, they are infected with *Mtb*, and (3) develop the characteristics of TB in humans [28,29]. Drawings from Servier Medical Art were used in some areas of the figure. Servier Medical Art by Servier is licensed beneath a CCA 3.0 Unported License (https://creativecommons.org/licenses/by/3.0/ (accessed on 7 May 2023).

A humanized mice model of TB has already been established. This model uses the human bone marrow, liver, and thymus (BLT). Human fetal liver, thymus tissue, and CD34+ fetal liver cells were engrafted into mice NOD-SCID/γc^null^. At 12 weeks following engraftment, excellent reconstitution was seen as evaluated by peripheral blood populations’ expression of the human CD45 pan leukocyte marker. The human leukocyte (CD45+) population included human T cells (CD3, CD4, CD8), natural killer cells, and monocyte/macrophages. Importantly, proliferative ability and the expression of effector molecules such IFNγ, granulysin, and perforin in response to positive stimuli were indicators of human T cells’ functional competence. Animals inoculated intranasally with *Mtb* experienced increasing lung infection and spread of the bacteria to the spleen and liver between two and eight weeks after infection. Organized granulomatous lesions, caseous necrosis, bronchial obstruction, and cholesterol deposit crystallization were signs of lung infection sites. Human T lymphocytes were arranged to the periphery of granulomas and dispersed throughout the lung, liver, and spleen at inflammation and bacteria growth areas [27,28]. A similar model produced by transplanted hematopoietic stem cells obtained from human fetal liver developed caseous necrotic granulomas [29] (Figure 4). These models could help study immunotherapies and new treatment development against TB as well as the immunopathology of the disease since they set the clinical characteristics of TB in humans.

## 4. Treatment of Pulmonary TB

Fortunately, there is an efficient pharmacological treatment for active TB, which consists of the administration of four different antibiotics: isoniazid (INH), rifampicin (RIF), streptomycin, and ethambutol for 4 to 6 months [30]. Due to the length of treatment, there are significant problems in compliance and toxicity. Treatment abandonment produces disease reactivation and, notably, the emergence of MDR, XDR, and TDR bacteria. Treatment of MDR-TB consists of administering up to eight second-line antibiotics for 12 and up to 18 months [31]. Therefore, this treatment is more complex, lengthy, costly, and toxic. For these reasons, it is crucial to design new therapeutic alternatives that allow shortening the therapy, one of which is to enhance the protective immune response, known as immunotherapy [10]. One modality of TB immunotherapy is the administration of recombinant adenoviruses that function as vectors of protective immunological factors.

### 4.1. Immunotherapy Based on Gene Therapy with Recombinant Adenovirus

Vectors based on viruses or viral particles are compact, effective, and have a high transmission potential. These viral vectors have undergone genetic engineering to overexpress antigens or genes and trigger immunological responses. As a result, they are employed in gene therapy as a delivery system [32]. Adenoviruses (Ad) belong to the Adenoviridae family, genus Mastadenovirus [33]. There are more than 103 human Ad serotypes. Type 5 is the most common and most studied as a gene transfer vector. Ad are non-enveloped viruses with icosahedral capsids of double-stranded DNA of 26 to 45 kb, encoding 40 proteins classified as early and late. The early genes E1A, E1B, E2, and E4 are expressed before DNA replication, and the late genes (L1–L5) encode the penton base, the hexon, the fibers that make up the capsid structure, and the proteins of the nucleoid (protein VII, protease). The Ad life cycle occurs within the infected cell’s nucleus, including DNA replication, gene expression, and virion formation. Ad receptors vary between species; the Ad and coxsackievirus receptor (CAR) represents the main entry route into the host cell. It belongs to the immunoglobulin superfamily. CAR is required for binding the Ad to the cell membrane [34,35]. CAR expression confers high tropism of Ad to hepatocytes, myoblasts, bronchial cells, and epithelial and endothelial cells [36]. Ad usually does not cause severe disease in immunocompetent persons.

#### 4.1.1. The Advantages of Ad as Genetic Vectors

Ad as genetic vectors have several advantages, such as high safety because they do not replicate. Ad vector genome maintenance as an episome in the nucleus lowers viral genomic DNA integration into the host’s genome, lowering the likelihood of insertional mutagenesis [32]. The production of the recombinant protein is transitory (for two or three weeks), but it is sufficient time to exert its biological activity. In addition, Ad are poorly immunogenic due to the absence of early genes 1 and 3, so they cause mild inflammation in the lungs and liver. These viral vectors have ligands for specific receptors highly expressed in the bronchial epithelium, making them ideal agents for treating lung diseases. The production of Ad is less expensive than recombinant proteins that are usually rapidly degraded and must be administered frequently. In contrast, the proteins produced by cells infected with recombinant Ad are constantly produced, maintaining their effectiveness for a more extended period after a single administration of the viral vector [37]. Furthermore, Ad vectors are frequently used as vaccine delivery systems because they effectively deliver foreign DNA into target cells and naturally trigger host immune responses [38].

#### 4.1.2. The Drawbacks of Ad as Genetic Vectors

Despite the advantages of using Ad as a genetic therapy and vaccine delivery system, some disadvantages that Ad present when used in this type of therapy must be considered. For instance, immune reactions to vectors and transgene products hinder consistent efficacy. Ad develop potent innate responses to the Ad capsid and genes expressed in the first-generation vectors [32]. The inflammatory reactions include vascular endothelial cells and platelet activation, inflammatory cytokine production, and macrophage cell death. There are reports of the death of patients with preexisting Ad immunity receiving the Ad-based gene therapy due to systemic inflammatory responses, later attributed to the administered virus [39]. The sequential administration of two or more antigenically different viruses is one strategy that might be employed to get around this problem. This strategy would guarantee that the second virus’s therapeutic benefits would not be hindered by the specific immunity that develops after the first virus is administered. Ad are flexible and tissue-specific. However, studies in animal models have shown that intravenous delivery of these viruses can cause severe liver injury [40]. Recently, it has been shown that a single inoculation with Ad5 induced body weight gain, produced hyperglycemia and hyperlipidemia, and generated morphological changes related to non-alcoholic fatty liver disease (NAFLD) in golden (Syrian) hamsters. These findings suggest that epidemiological research, particularly studies on the long-term impact of gene therapy, is required to assess the role of Ad as a risk factor for chronic liver and cardiovascular illnesses [41].

#### 4.1.3. Construction of Recombinant Ad

**Figure 5:**
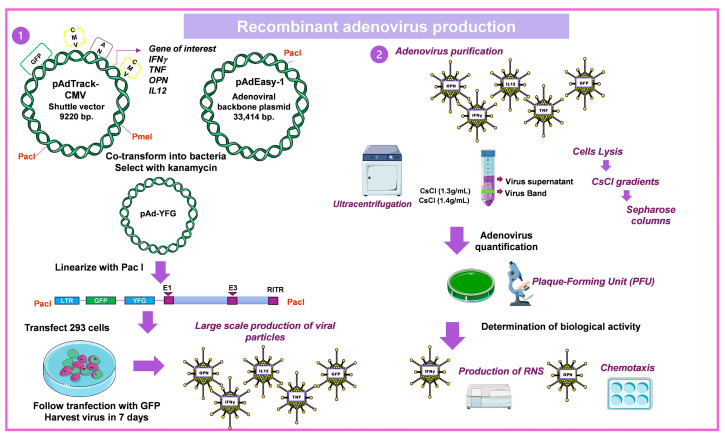
Construction, production, and purification of recombinant adenovirus. The structure of recombinant adenovirus was created following the methodology established by He et al., 1998 [42]. (1) The gene of interest is cloned into the pAdtrack-CMV plasmid (9220 bp) that is linearized with the enzyme PmeI and cotransformed into *E. coli* BJ5183 with the adenoviral plasmid pAdEasy-1 (33,414 bp) which occurs between the left- and right-arm homologous recombination regions between pAdtrack-CMV and pAdEasy-1. Recombinant adenoviruses are selected for their resistance to kanamycin, and recombination is confirmed by restriction enzyme analysis. The recombinant plasmid linearized with PacI is transfected into 293 packaging cells. (2) They are produced on a large scale, lysed by heat shock, and purified by CsCl gradients and Sepharose columns. Adenovirus is quantified by determining plaque-forming units (PFU) and performing a specific assay to verify its biological activity. For example, for AdIFNγ, its ability to form reactive nitrogen intermediates (RNS) was evaluated [43], and for AdOPN, a chemotaxis assay was performed [44]. Some parts of the figure were adapted from the work of He et al., 1998 [41], and permission is not needed to use it. Drawings from Servier Medical Art were used in some areas of the figure. Servier Medical Art by Servier is licensed beneath a CCA 3.0 Unported License (https://creativecommons.org/licenses/by/3.0/ (accessed on 7 May 2023)).

**Figure 6:**
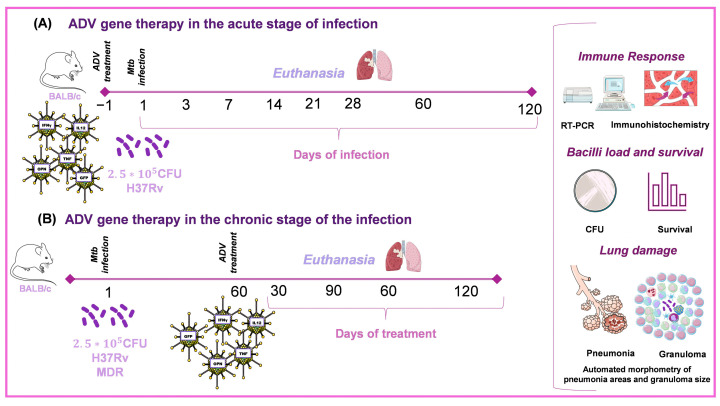
Ad gene therapy in the early or late stage of progressive TB. (**A**) One day before infection, 8-week-old male mice are treated with recombinant Ad. Then, animals are infected with a high dose (250,000 CFU) of the H37Rv reference drug sensible strain or the MDR clinical isolate. (**B**) 8-week-old male mice are infected via IT with 250,000 CFU of the H37Rv-type strain or the MDR clinical isolate. At day 60 post-infection, groups of mice are treated with only one dose of recombinant Ad or in combination with first- or second-line antibiotics [43]. Drawings from Servier Medical Art were used in some areas of the figure. Servier Medical Art by Servier is licensed beneath a CCA 3.0 Unported License (https://creativecommons.org/licenses/by/3.0/ (accessed on 7 May 2023)).

The steps followed to build and apply the Ad vectors are: first, make the Ad vector with the gene of interest [42], then determine its concentration and biological activity evaluated in in vitro assays and follow by determining the kinetics of gene expression of the recombinant protein in healthy animals after administering the Ad directly into the lung by intratracheal injection, in different doses to determine the most efficient and the course time of expression of the transfected gene, as well as to analyze the produced histological damage [43] (Figure 5). Finally, the therapeutic effect of Ad in the different murine models of TB is evaluated (Figure 6) [43].

### 4.2. Recombinant Adenovirus Therapy in Different Experimental Models of TB

Knowledge of the protective and non-protective immune response during TB allows us to intercede therapeutically during the disease, providing protective factors when these are little produced or suppressing deleterious factors that block protective immunity [13]. This intervention in the immune response allows efficient elimination of the infectious agent and is the basis of immunotherapy [10]. This type of therapeutic intervention is described below using Ad vectors that carry genes that encode essential immunological factors in the control of growth and eradication of mycobacteria, which constitutes gene therapy to treat active TB and prevent the reactivation of latent TB and transmission or contagion to close contacts of TB patients. In some cases, a single administration of the recombinant adenovirus is required to obtain the therapeutic effect. In other instances of several applications, the intratracheal route has been used in most experiments, but the intranasal route is also a minimally invasive route with great therapeutical potential [45].

#### 4.2.1. AdIFNγ

IFNγ is a crucial cytokine in the control of TB since it activates macrophages and induces the expression of class II histocompatibility complex molecules, enhancing the presentation of antigens to T cells. It also induces other pro-inflammatory cytokines, such as TNFα in macrophages, its receptor, and the enzyme iNOS. Therefore, IFNγ contributes to efficiently controlling TB [46]. On day 60 post-infection, when TB is advanced because it produces extensive pneumonia with a high bacillary load, a single dose of IFNγ recombinant adenovirus (AdIFNγ) was administered to mice infected with the drug-sensitive reference strain H37Rv or with the MDR CIBIN99 strain; with this treatment, there was a significant decrease in the bacterial load and pneumonia in both strains. When AdIFNγ was administered in combination with the conventional treatment (INH, PIR, RIF), the bacterial load and pneumonia were significantly reduced compared to animals treated with antibiotics alone, indicating that gene therapy substantially shortens the time of antibiotic therapy [43]. Interestingly, Plasmids bearing murine IFNγ have shown similar results in a model of TB using male Swiss mice infected with a low-dose aerosol *Mtb*. When mice were given roughly five ng of a plasmid containing the mouse IFNγ gene once a week for four weeks by inhalation route, there was a considerable decrease in the bacterial burden and an improvement in the histology and gross morphology of the lungs [47,48].

#### 4.2.2. AdIL12

Interleukin 12 (IL12) is produced by macrophages, NK cells, and dendritic cells in response to pathogens or by stimulating other cytokines [49]. IL-12 converts immature T cells to effector T cells, promotes dendritic cell migration, and inhibits anti-inflammatory cytokines. However, its most prominent activity is activating the Th1 response [49], making it an ideal candidate for evaluating its therapeutic effect in our TB models. AdIL-12 was administered twice in the progressive TB model to evaluate its prophylactic and therapeutic effects. AdIL-12 was administered one day before infection with the H37Rv reference strain to evaluate its prophylactic effect. Compared to control animals that received empty Ad, mice treated with AdIL-12 showed a significant decrease in bacterial load, less pneumonia, and higher expression of IFNγ, TNFα, and iNOS [25]. AdIL-12 was also tested in the cohoused model. A single dose of AdIL12 was administered intratracheally to healthy mice living with mice infected with a highly virulent and transmissible mycobacterial strain (Beijing strain 9001000). All living mice were not infected [45]. In the study of the therapeutic activity of AdIL-12 in progressive TB, four intranasal doses were administered in the late phase of the infection (60 days). This treatment significantly decreased the bacillary load [45].

#### 4.2.3. AdGM/CSF

One of the characteristics of our model of progressive TB is the delay in the activation of dendritic cells, whereby the bacteria can better adapt without being eliminated during early infection [50]. The most efficient factor for activating dendritic cells is the granulocyte/macrophage colony stimulated growth factor (GM/CSF). For this reason, the prophylactic effect of AdGM/CSF was evaluated, administering them one day before infection with the drug-sensitive strain of *Mtb* H37Rv [51]. With this treatment, flow cytometry showed a significant advance in the presence of activated dendritic cells, which prevented the increase in the bacterial load in contrast with control mice that received the empty Ad. AdGM/CSF administration also prevented pneumonia, in addition to inducing an increase in the number and size of granulomas, which in this model indicates good protection. In our model of progressive TB, during the advanced phase, when there is extensive pneumonia and a high bacterial load, there is also a marked decrease in dendritic cells [50] and GM/CSF [51]. In this phase, a single dose of AdGM/CSF on day 60 of infection significantly decreased the bacillary load compared to mice receiving empty Ad. In animals infected with drug-susceptible bacteria or MDR strain, AdGM/CSF also shortened treatment time when administered with antibiotics [52].

Latent TB affects a quarter of the world’s population, constituting the largest reservoir of *Mtb*. It is estimated that 10% of these infected subjects may suffer reactivation, developing active TB due to the coexistence of diseases that depress the immune system, such as diabetes mellitus, chronic kidney diseases, and HIV infection, among others [6]. For these reasons, an essential aspect is to develop therapies that prevent the reactivation of latent TB. In a murine model similar to latent TB, we evaluated AdGM/CSF for its ability to prevent TB from being reactivated by administering corticosterone, a potent immunosuppressant. With the administration of AdGM/CSF in a single dose in mice with latent TB subsequently subjected to immunosuppression induced by the administration of corticosterone for one month, it was observed that the treatment with AdGM/CSF prevented the growth of bacteria and the spread of pneumonia, which demonstrates that this treatment prevents the reactivation of experimental latent TB [51].

TB is a highly contagious disease, so close convivients of patients with active TB are at high risk of becoming infected and developing progressive TB. Our model of transmissibility reproduces this condition; mice with active TB induced by infection with the reference strain H37Rv or with a hypervirulent and highly contagious clinical isolate living with a group of healthy mice in the same microisolator produced infection of this group [25]. In this experimental model, we tested the efficiency of AdGM/CSF, comparing normal mice treated with AdGM/CSF with negative control mice that received empty Ad or positive control that corresponded to healthy mice treated with INH, which is the treatment to prevent infection in people that is in close contact with active TB patients. The results showed that the administration of AdGM/CSF prevented healthy mice from being infected with H37Rv or with the hypervirulent clinical isolate Beijing 9001000 strain after two months of living with sick mice, making this treatment even better than INH administration [51].

#### 4.2.4. AdOPN

Osteopontin (OPN) is a protein that participates in biomineralization, wound healing, and inflammation. Macrophages and T cells produce OPN and induce chemotaxis, dendritic cell maturation, and Th-1 cell activation [53]. AdOPN was administered in the late phase of progressive TB in mice infected with the MDR strain of *Mtb* CIBIN99. The therapy with AdOPN produced a significant decrease in bacterial load and pneumonia, with an increase in granulomas. AdOPN induced more considerable expression of IL12, IFNγ, TNFα, and IL17, which efficiently controlled the mycobacterial infection [44].

#### 4.2.5. Ad Antimicrobial Peptides

In the primary *Mtb* infection, bacteria are eliminated efficiently, and only 5% of the cases develop active TB. The exact cause of this is unknown, but it is likely to be the efficiency of innate immunity [3]. *Mtb* enters through the respiratory system, and the first line of defense is the respiratory epithelium and alveolar macrophages, whose main protective activities are phagocytosis and the production of antimicrobial peptides (AMP) such as defensins and cathelicidins [15]. AMPs are small, cationic molecules that form pores in the membranes of microorganisms, causing lysis. They are also chemotactic regulators of immune cells, so macrophage-activating molecules and PAMs have great potential in immunotherapy [54]. High levels of the antimicrobial peptides PAMs beta-defensin (βDEF) and cathelicidin (LL37) are also of great relevance for maintaining mycobacteria in a latent state [55].

In our murine models of latent and progressive pulmonary TB, we observed that the abundance of classic activated M1 macrophages and AMPs contributes to the initial infection control [19,20,56]. In contrast, in the advanced stage of TB they decrease, which contributes to increasing bacterial proliferation and tissue damage (pneumonia). During this progressive phase, we studied the therapeutic effect of recombinant adenoviruses that express AMPs [57]. In all these studies, the bacillary load was significantly reduced in mice with drug-sensitive TB and MDR, and the time on antibiotic treatment was shortened [57]. Moreover, the recombinant adenoviruses encoding β defensin-3 and LL37 decreased bacterial load and pneumonia in the chronic infection model similar to latent TB [58].

#### 4.2.6. Ad TNFα

The pro-inflammatory cytokine TNFα is critical for maintaining the latency state, as demonstrated by the reactivation of latent TB in patients with rheumatoid arthritis receiving neutralizing antibodies to TNFα [59]. Due to the importance of TNFα in keeping latent TB, we used the corresponding model and the application of AdTNFα that efficiently prevented an increase in the pulmonary bacterial load in animals subjected to immunosuppression with glucocorticoids compared to the control [58]. A significant effect of AdTNFα in reducing pulmonary bacillary loads and pneumonia, as well as an adjuvant of second-line antibiotics, was recently observed in the model of progressive pulmonary TB induced by MDR *Mtb* (manuscript in preparation).

#### 4.2.7. Ad IL-23

IL-23 is a member of the IL-12 cytokine family and participates in the host defense against *Mtb.* In a murine model of pulmonary TB using C57BL/6 mice infected with *Mtb* strain H37Rv, administering an AdIL-23 before infection decreased the lung bacilli load at days 14, 21, and 28 post-infection [59]. The treatment was also effective in decreasing lung damage and inflammation. Moreover, AdIL-23 pre-treatment increased the number of T CD4^+^ cells and the levels of IFNγ and IL-17. This study showed that single-dose vector-mediated pulmonary IL-23 gene delivery was safe and effective in limiting the growth of *Mtb* in the lungs [60].

### 4.3. Candidate Vaccine for TB Based on Adenovirus

Vaccines are an effective defense against various infections and can stop the spread of infectious diseases [37]. The Ad vector-based vaccines are promising as possible vaccine vectors for infectious diseases such as TB because they have several characteristics, including high transduction efficiencies and high titer production [61,62]. We describe some of the Ad vector-based TB vaccines in this review section.

#### 4.3.1. AdHu5Ag85A

Phase I clinical studies have been conducted with a recombinant, replication-deficient human Ad5 vector expressing the *Mtb* antigen, Ag85A (AdHu5Ag85A). Ad5Ag85A vaccination either intranasally or intramuscularly activated antigen-specific CD4^+^ and CD8^+^ T cells in mice [63,64]. However, only intranasal administration—not intramuscular administration—of the vaccine produced long-lived CD8^+^ T cells in the airway lumen, suggesting that the route of vaccination (intranasally or intramuscularly) may be able to influence the different immune profiles. In multiple animal models, such as calves, guinea pigs, and goats, the Ad5Ag85A vaccine was examined using various delivery methods, including intramuscular, intradermal, and endobronchial. According to reports, the vaccination is safe and effective at preventing the growth of several mycobacterial strains, including *M. bovis*, *Mtb*, and *M. caprae* [63,64,65,66].

#### 4.3.2. ChAdOx185A-MVA85A

Based on a chimpanzee Ad vector producing *Mtb* antigen 85A (Ag85A), ChAdOx185A-MVA85A expresses *Mtb* antigen 85A. In preclinical trials, BCG-ChAdOx185A-MVA85A was safe and protective when administered to mice compared to BCG control alone and combined with modified vaccinia Ankara, also expressing Ag85A (MVA85A). ChAdOx1.85A and MVA85A, given mucosal or systemically, elicited potent immune responses and increased the protective effectiveness of BCG [67]. ChAdOx185A was well accepted, safe, and immunogenic for healthy UK adults’ treatment when added to BCG or MVA85A [68]. Another Phase I clinical research project has been carried out to see if the immunogenicity of the vaccine relies on the delivery methods, i.e., aerosol versus intramuscular in healthy adult people. ChAdOx1-85A vaccination via aerosol administration increased mucosal cellular responses, particularly IFNγ/IL-17^+^ CD4^+^ T cells, while immunization via intramuscular injection increased systemic cellular and humoral responses. Fatigue and headache were the most frequently reported adverse symptoms [69].

#### 4.3.3. AERAS-402

AERAS-402 is based on the replication-deficient Ad35 and includes *Mtb* antigens TB10.4, 85A, and 85B. These *Mtb* antigens are immunogenic and strong T-cell epitopes. AERAS-402 was safe in phase I clinical studies among individuals from India, a country with a high TB prevalence, who had received the BCG vaccine. Immune profiling investigations revealed the release of pro-inflammatory cytokines such as IFNγ, TNFα, and IL-2 and the development of a strong polyfunctional CD8^+^ T cell in response to Ag85B [70]. According to another study, AERAS-402 was safe and immunogenic for healthy infants receiving the BCG vaccine [71].

### 4.4. Other Virus-Based Therapies in TB

In addition to Ad, other virus-based treatments have been used in TB. In this section, we briefly describe some of them.

#### 4.4.1. MVA/IL-15/5Mtb

A vectored vaccine utilizing the modified virus Ankara (MVA) strain of vaccinia virus expressing the antigens ESAT6, Ag85A, Ag85B, HSP65, and Mtb39A of *Mtb* together with interleukin-15 (MVA/IL-15/5Mtb) induced comparable CD4^+^ T cell and greater CD8^+^ T cell and antibody responses against *Mtb* in vaccinated BALB/c and C57BL/6 mice in a direct comparison with the BCG vaccine. It conferred protection against an aerogenic challenge of *Mtb* [72].

#### 4.4.2. VSVAg85A

A brand-new TB vaccine that expresses Ag85A using the vesicular stomatitis virus (VSV) as a viral vector system (VSVAg85A) was administered to mice intramuscularly (IM) or intranasally (IN). Both immunogenic immunization methods produced unique T-cell profiles, but only intranasal administration led to a protective mucosal T-cell response in response to a pulmonary Mtb challenge. Therefore, experiments were conducted to determine whether VSVAg85A might be utilized as a mucosal booster for parenteral priming by an adenoviral TB vaccine (AdAg85A). After AdAg85A priming, VSVAg85A immunization significantly increased antigen-specific T-cell responses in the airway lumen while enhancing immunological activation in the systemic compartment. This resulted in a noticeably higher level of protective efficacy against lung MTB challenge than either vaccine administered alone. Because of this, this research shows that VSV is a good candidate for a heterologous viral prime-boost vaccination regimen against intracellular bacterial infection [73].

#### 4.4.3. Mycobacteriophage D29

By preexposure prophylactically administering active aerosolized antiTB bacteriophage D29 to the lungs, protection against *Mtb* infection was examined. Using a nose-only inhalation device improved with a dosage simulation technique, an average bacteriophage concentration of about 1 PFU/alveolus was attained in the lungs of mice. The mice were given either a low dose of *Mtb* strain H37Rv aerosol to the lungs (about 50 to 100 CFU) or an ultralow dosage (roughly 5 to 10 CFU) within 30 min after the bacteriophage delivery. Bacteriophage aerosol pre-treatment significantly reduced the amount of *Mtb* in mouse lungs at 24 h and 3 weeks after exposure, indicating a preventative impact [74,75].

#### 4.4.4. TB/FLU-01L

A replication-deficient influenza A virus expressing the ESAT-6 antigen comprises the live vector vaccine TB/FLU-01L. Preclinical studies in C57BL/6 mice, BALB/c mice, ferrets, and non-human primates demonstrated that TB/FLU-01L vaccine administered intranasally or sublingually was safe and immunogenic (innate and adaptive cellular immune response). Moreover, TB/FLU-01L presented a substantial immunotherapeutic effect in mice with preestablished TB infection and had a synergistic effect with different treatment schemes. When given intranasally or sublingually to healthy people who had received the BCG vaccine, the TB/FLU-01L TB vaccine was found to be safe and well-tolerated in a Phase clinical trial. In this investigation, no unfavorable reaction to viral carrier particles was seen, and 70% of the vaccinated participants were able to react to *Mtb* antigens [76].

#### 4.4.5. TB/FLU-04L

Using an attenuated influenza A virus vector containing two mycobacterium antigens, Ag85A and ESAT-6, a novel intranasal TB vaccine candidate, TB/FLU-04L, was created. An *Mtb*-specific Th1 immune response was generated in C57BL/6 mice or cynomolgus macaques after intranasal administration of the TB/FLU-04L vaccine candidate. When administered as part of a “prime-boost” strategy, a single immunization with TB/FLU-04L vaccine in mice demonstrated comparable levels of protection to BCG. It dramatically improved the protective effect of BCG [77].

#### 4.4.6. RhCMV/TB

Using a rhesus cytomegalovirus vector to express *Mtb* antigens (ESAT-6, Ag85A, Ag85B, Rv3407, Rv1733, Rv2626, Rpf A, Rpf C, and Rpf D), scientists have created the recombinant vaccine RhCMV/TB. Subcutaneous vaccination of rhesus macaques generated effector-differentiated CD4^+^ and CD8^+^ memory T-cell responses against *Mtb* antigens. It reduced by 68% *Mtb* infection when rhesus macaques were exposed to *Mtb* (Erdman strain) one year after vaccination [78].

#### 4.4.7. rLCMV-Based Mtb Vaccine

Neonatal and adult mice were used to test the replication-deficient lymphocytic choriomeningitis virus (rLCMV)-based vaccine vector producing *Mtb* antigens Ag85B and TB10.4 (Ag85B-TB10.4). After delivery, it was noted that this vaccine could produce heterogeneous CD4^+^, and CD8^+^ T-cell populations, and lung damage when infected with *Mtb* was reduced. CD8^+^ but not CD4^+^ T cells were efficiently boosted upon vector re-vaccination. According to this research, rLCMV may be helpful for adult and/or neonatal vaccination programs against pulmonary TB [79].

## 5. Perspectives

TB continues to be a public health problem. The emergence of MDR, XDR, and TDR strains urgently requires new therapies to combat this disease. Furthermore, the inefficiency of BCG in controlling pulmonary TB contributes to the inefficient control of this disease. In this sense, using viral vectors as gene therapy and developing vaccines has contributed crucially to developing new therapeutic and vaccine strategies for this disease. Although most of the studies on gene therapy in TB were carried out in the preclinical phase, the results obtained, mainly in murine models, are encouraging and demonstrate that they are adequate for controlling TB so that clinical studies can be carried out soon. Due to the complicated immunopathological interaction in TB, many immunotherapeutic strategies can still be implemented in this terrible disease. For example, Ad (AdTNFα and AdGM/CSF) combinations could give synergistic results, given that their separate use has generated excellent results in prophylactic and therapeutic therapy. Another proposal is using Ad mediated by siRNA with the specific target IL10. This anti-inflammatory cytokine impairs the control of TB in the chronic phase. Many additional viral vectors are under preclinical testing and are in various stages of clinical studies for treating hereditary and acquired disorders. Viral vector gene therapy has proven successful in treating or even curing diseases for which there were no or only ineffective medicines at the time.

In the case of TB vaccines based on viral vectors, as we have described, many are in the clinical phase, which demonstrates their efficiency and safety in the control of TB, in addition to the fact that Ad-based COVID-19 vaccines are used safely in the general population, and several medications using viral vector-based gene therapy have recently been given the green light for commercialization. Therefore, we are getting closer to testing Ad in patients with active TB as a therapeutic alternative to conventional pharmacological treatments and vaccination, reducing treatment time and collateral damage as well as the control of and reduction in the spread of the disease.

## 6. Conclusions

In searching for new TB treatments, immunotherapy has emerged as a viable alternative for treating MDR- or even extensively drug-resistant TB. The initial strategy in the design of recombinant Ad used as immunotherapy was to choose immunological factors (cytokines, PAM) that will activate macrophages and T lymphocytes, polarizing them towards the M1 and Th1 profile, respectively, to control mycobacterial growth. Our experimental results show that gene therapy in TB can be beneficial to shorten treatment with conventional and second-line TB chemotherapy. The advantages of its use in pulmonary TB are the tropism of Ad to the respiratory epithelium and alveolar macrophages and the fact that a single dose was efficient. Our results also show that some Ad can be used in prophylactic experiments, such as preventing latent TB reactivation or bacterial transmission. In addition, vaccines based on viral vectors against TB have demonstrated significant protective effects. These results support one of the main goals of the STOP-TB strategy by identifying novel treatments for decreasing the global burden of TB, generating vaccines against TB, and preventing the spread of this disease.

## Data Availability

Data sharing is not applicable.

## References

[1] Rahlwes K.C., Dias B.R.S., Campos P.C., Alvarez-Arguedas S., Shiloh M.U. (2023). Pathogenicity and virulence of Mycobacterium tuberculosis. Virulence.

[2] Ojo O.O., Nadarajah S., Kebe M. (2023). Integer time series models for tuberculosis in Africa. Sci. Rep..

[3] Sakamoto K. (2012). The Pathology of Mycobacterium tuberculosis Infection. Vet. Pathol..

[4] Poladian N., Orujyan D., Narinyan W., Oganyan A.K., Navasardyan I., Velpuri P., Chorbajian A., Venketaraman V. (2023). Role of NF-κB during Mycobacterium tuberculosis Infection. Int. J. Mol. Sci..

[5] O’Garra A., Redford P.S., McNab F.W., Bloom C.I., Wilkinson R.J., Berry M.P.R. (2013). The Immune Response in Tuberculosis. Annu. Rev. Immunol..

[6] World Health Organization (2021). Global Tuberculosis Report 2020: Synopsis [Global Tuberculosis Report 2020: Executive Summary].

[7] Schrager L.K., Vekemens J., Drager N., Lewinsohn D.M., Olesen O.F. (2020). The status of tuberculosis vaccine development. Lancet Infect. Dis..

[8] Pandit R., Singh P.K., Kumar V. (2015). Natural Remedies against Multi-Drug Resistant Mycobacterium tuberculosis. J. Tuberc. Res..

[9] Hameed H.M.A., Islam M.M., Chhotaray C., Wang C., Liu Y., Tan Y., Li X., Tan S., Delorme V., Yew W.W. (2018). Molecular targets related drug resistance mechanisms in MDR-, XDR-, and TDR-Mycobacterium tuberculosis strains. Front. Cell. Infect. Microbiol..

[10] Ramos-Espinosa O., Islas-Weinstein L., Peralta-Álvarez M.P., López-Torres M.O., Hernández-Pando R. (2018). The use of immunotherapy for the treatment of tuberculosis. Expert Rev. Respir. Med..

[11] Wirth T., Parker N., Ylä-Herttuala S. (2013). History of gene therapy. Gene.

[12] Kreppel F., Kochanek S. (2008). Modification of adenovirus gene transfer vectors with synthetic polymers: A scientific review and technical guide. Mol. Ther..

[13] De Martino M., Lodi L., Galli L., Chiappini E. (2019). Immune Response to Mycobacterium tuberculosis: A Narrative Review. Front. Pediatr..

[14] Carabalí-Isajar M.L., Rodríguez-Bejarano O.H., Amado T., Patarroyo M.A., Izquierdo M.A., Lutz J.R., Ocampo M. (2023). Clinical manifestations and immune response to tuberculosis. World J. Microbiol. Biotechnol..

[15] Rivas-Santiago C.E., Hernández-Pando R., Rivas-Santiago B. (2013). Immunotherapy for pulmonary TB: Antimicrobial peptides and their inducers. Immunotherapy.

[16] Hernandez-Pando R., Orozco H., Aguilar D. (2009). Factors that deregulate the protective immune response in tuberculosis. Arch. Immunol. Ther. Exp..

[17] Rook G.A.W. (2007). Endocrine and cytokine responses in humans with pulmonary tuberculosis. Brain. Behav. Immun..

[18] Hernández-Pando R., Orozcoe H., Sampieri A., Pavón L., Velasquillo C., Larriva-Sahd J., Alcocer J.M., Madrid M. (1996). V Correlation between the kinetics of Th1, Th2 cells and pathology in a murine model of experimental pulmonary tuberculosis. Immunology.

[19] Hernandez-Pando R., Orozco E.H., Arriaga K., Sampieri A., Larriva-Sahd J., Madrid-Marina V. (1997). Analysis of the local kinetics and localization of interleukin-1α, tumour necrosis factor-α and transforming growth factor-β, during the course of experimental pulmonary tuberculosis. Immunology.

[20] Rivas-Santiago B., Sada E., Tsutsumi V., Aguilar-Léon D., Contreras J.L., Hernández-Pando R. (2006). Β-Defensin Gene Expression During the Course of Experimental Tuberculosis Infection. J. Infect. Dis..

[21] Sharma S.K., Mohan A., Kohli M. (2021). Extrapulmonary tuberculosis. Expert Rev. Respir. Med..

[22] Rodriguez-Takeuchi S.Y., Renjifo M.E., Medina F.J. (2019). Extrapulmonary tuberculosis: Pathophysiology and imaging findings. Radiographics.

[23] Hernandez-Pando R., Orozco H., Arriaga K., Pavön L., Rook G. (2000). Treatment with BB-94, A broad spectrum inhibitor of zinc-dependent metalloproteinases, causes deviation of the cytokine profile towards Type-2 in experimental pulmonary tuberculosis in Balb/c mice. Int. J. Exp. Pathol..

[24] Arriaga A.K., Orozco E.H., Aguilar L.D., Rook G.A.W., Hernández Pando R. (2002). Immunological and pathological comparative analysis between experimental latent tuberculous infection and progressive pulmonary tuberculosis. Clin. Exp. Immunol..

[25] Marquina-Castillo B., García-García L., Ponce-De-León A., Jimenez-Corona M.E., Bobadilla-Del Valle M., Cano-Arellano B., Canizales-Quintero S., Martinez-Gamboa A., Kato-Maeda M., Robertson B. (2009). Virulence, immunopathology and transmissibility of selected strains of Mycobacterium tuberculosis in a murine model. Immunology.

[26] Walsh N.C., Kenney L.L., Jangalwe S., Aryee K.E., Greiner D.L., Brehm M.A., Shultz L.D. (2017). Humanized Mouse Models of Clinical Disease. Annu. Rev. Pathol. Mech. Dis..

[27] Calderon V.E., Valbuena G., Goez Y., Judy B.M., Huante M.B., Sutjita P., Johnston R.K., Estes D.M., Hunter R.L., Actor J.K. (2013). A Humanized Mouse Model of Tuberculosis. PLoS ONE.

[28] Nusbaum R.J., Calderon V.E., Huante M.B., Sutjita P., Vijayakumar S., Lancaster K.L., Hunter R.L., Actor J.K., Cirillo J.D., Aronson J. (2016). Pulmonary Tuberculosis in Humanized Mice Infected with HIV-1. Sci. Rep..

[29] Arrey F., Löwe D., Kuhlmann S., Kaiser P., Moura-Alves P., Krishnamoorthy G., Lozza L., Maertzdorf J., Skrahina T., Skrahina A. (2019). Humanized mouse model mimicking pathology of human tuberculosis for in vivo evaluation of drug regimens. Front. Immunol..

[30] Suárez I., Fünger S.M., Kröger S., Rademacher J., Fätkenheuer G., Rybniker J. (2019). The Diagnosis and Treatment of Tuberculosis. Dtsch. Arztebl. Int..

[31] Anderson L.F., Tamne S., Watson J.P., Cohen T., Mitnick C., Brown T., Drobniewski F., Abubakar I. (2013). Treatment outcome of multi-drug resistant tuberculosis in the United Kingdom: Retrospective-prospective cohort study from 2004 to 2007. Eurosurveillance.

[32] Appaiahgari M.B., Vrati S. (2015). Adenoviruses as gene/vaccine delivery vectors: Promises and pitfalls. Expert Opin. Biol. Ther..

[33] Harrach B., Tarján Z.L., Benkő M. (2019). Adenoviruses across the animal kingdom: A walk in the zoo. FEBS Lett..

[34] Loustalot F., Kremer E.J., Salinas S. (2016). Membrane Dynamics and Signaling of the Coxsackievirus and Adenovirus Receptor. Int. Rev. Cell Mol. Biol..

[35] Hidaka C., Milano E., Leopold P.L., Bergelson J.M., Hackett N.R., Finberg R.W., Wickham T.J., Kovesdi I., Roelvink P., Crystal R.G. (1999). CAR-dependent and CAR-independent pathways of adenovirus vector-mediated gene transfer and expression in human fibroblasts. J. Clin. Investig..

[36] Kulanayake S., Tikoo S.K. (2021). Adenovirus core proteins: Structure and function. Viruses.

[37] Garbuglia A.R., Minosse C., Del Porto P. (2022). mRNA- and Adenovirus-Based Vaccines against SARS-CoV-2 in HIV-Positive People. Viruses.

[38] Srivastava S., Dey S., Mukhopadhyay S. (2023). Vaccines against Tuberculosis: Where Are We Now?. Vaccines.

[39] Shirley J.L., de Jong Y.P., Terhorst C., Herzog R.W. (2020). Immune Responses to Viral Gene Therapy Vectors. Mol. Ther..

[40] Young L.S., Mautner V. (2001). The promise and potential hazards of adenovirus gene therapy. Gut.

[41] Montes-Galindo D.A., Espiritu-Mojarro A.C., Melnikov V., Moy-López N.A., Soriano-Hernandez A.D., Galvan-Salazar H.R., Guzman-Muñiz J., Guzman-Esquivel J., Martinez-Fierro M.L., Rodriguez-Sanchez I.P. (2019). Adenovirus 5 produces obesity and adverse metabolic, morphological, and functional changes in the long term in animals fed a balanced diet or a high-fat diet: A study on hamsters. Arch. Virol..

[42] He T.C., Zhou S., Da Costa L.T., Yu J., Kinzler K.W., Vogelstein B. (1998). A simplified system for generating recombinant adenoviruses. Proc. Natl. Acad. Sci. USA.

[43] Mata-Espinosa D.A., Mendoza-Rodríguez V., Aguilar-León D., Rosales R., López-Casillas F., Hernández-Pando R. (2008). Therapeutic effect of recombinant adenovirus encoding interferon-γ in a murine model of progressive pulmonary tuberculosis. Mol. Ther..

[44] Hernández-Bazán S., Mata-Espinosa D., Lozano-Ordaz V., Ramos-Espinosa O., Barrios-Payán J., López-Casillas F., Pando R.H. (2022). Immune Regulatory Effect of Osteopontin Gene Therapy in a Murine Model of Multidrug Resistant Pulmonary Tuberculosis. Hum. Gene Ther..

[45] Mata-Espinosa D.A., Francisco-Cruz A., Marquina-Castillo B., Barrios-Payan J., Ramos-Espinosa O., Bini E.I., Xing Z., Hernández-Pando R. (2019). Immunotherapeutic effects of recombinant adenovirus encoding interleukin 12 in experimental pulmonary tuberculosis. Scand. J. Immunol..

[46] Flynn J.A.L., Chan J., Triebold K.J., Dalton D.K., Stewart T.A., Bloom B.R. (1993). An essential role for interferon γ in resistance to mycobacterium tuberculosis infection. J. Exp. Med..

[47] Bharti R., Srivastava A., Roy T., Verma K., Reddy D.V.S., Shafi H., Verma S., Raman S.K., Singh A.K., Singh J. (2020). Transient Transfection of the Respiratory Epithelium with Gamma Interferon for Host-Directed Therapy in Pulmonary Tuberculosis. Mol. Ther. Nucleic Acids.

[48] Bharti R., Roy T., Verma S., Reddy D.V.S., Shafi H., Verma K., Raman S.K., Pal S., Azmi L., Singh A.K. (2022). Transient, inhaled gene therapy with gamma interferon mitigates pathology induced by host response in a mouse model of tuberculosis. Tuberculosis.

[49] Cheng E.M., Tsarovsky N.W., Sondel P.M., Rakhmilevich A.L. (2022). Interleukin-12 as an in situ cancer vaccine component: A review. Cancer Immunol. Immunother..

[50] García-Romo G.S., Pedroza-González A., Aguilar-León D., Orozco-Estevez H., Lambrecht B.N., Estrada-Garcia I., Flores-Romo L., Hernández-Pando R. (2004). Airways infection with virulent Mycobacterium tuberculosis delays the influx of dendritic cells and the expression of costimulatory molecules in mediastinal lymph nodes. Immunology.

[51] Francisco-Cruz A., Mata-Espinosa D., Estrada-Parra S., Xing Z., Hernández-Pando R. (2013). Immunotherapeutic effects of recombinant adenovirus encoding granulocyte-macrophage colony-stimulating factor in experimental pulmonary tuberculosis. Clin. Exp. Immunol..

[52] Francisco-Cruz A., Mata-Espinosa D., Ramos-Espinosa O., Marquina-Castillo B., Estrada-Parra S., Xing Z., Hernández-Pando R. (2016). Efficacy of gene-therapy based on adenovirus encoding granulocyte-macrophage colony-stimulating factor in drug-sensitive and drug-resistant experimental pulmonary tuberculosis. Tuberculosis.

[53] Wang K.X., Denhardt D.T. (2008). Osteopontin: Role in immune regulation and stress responses. Cytokine Growth Factor Rev..

[54] Cruz J., Ortiz C., Guzmán F., Fernández-Lafuente R., Torres R. (2014). Antimicrobial Peptides: Promising Compounds Against Pathogenic Microorganisms. Curr. Med. Chem..

[55] Rivas-Santiago B., Contreras J.C.L., Sada E., Hernández-Pando R. (2008). The potential role of lung epithelial cells and β-defensins in experimental latent tuberculosis. Scand. J. Immunol..

[56] Castañeda-Delgado J., Hernández-Pando R., Serrano C.J., Aguilar-León D., León-Contreras J., Rivas-Santiago C., Méndez R., González-Curiel I., Enciso-Moreno A., Rivas-Santiago B. (2010). Kinetics and cellular sources of cathelicidin during the course of experimental latent tuberculous infection and progressive pulmonary tuberculosis. Clin. Exp. Immunol..

[57] Ramos-Espinosa O., Mata-Espinosa D., Francisco-Cruz A., López-Torres M.O., Hernández-Bazán S., Barrios-Payán J., Marquina-Castillo B., Carretero M., del Río M., Hernández-Pando R. (2021). Immunotherapeutic effect of adenovirus encoding antimicrobial peptides in experimental pulmonary tuberculosis. J. Leukoc. Biol..

[58] Ramos-Espinosa O., Hernández-Bazán S., Francisco-Cruz A., Mata-Espinosa D., Barrios-Payán J., Marquina-Castillo B., López-Casillas F., Carretero M., del Río M., Hernández-Pando R. (2016). Gene therapy based in antimicrobial peptides and pro-inflammatory cytokine prevents reactivation of experimental latent tuberculosis. Pathog. Dis..

[59] Keane J., Gershon S., Wise R.P., Mirabile-Levens E., Kasznica J., Schwieterman W.D., Siegel J.N., Braun M.M. (2001). Tuberculosis associated with infliximab, a tumor necrosis factor alpha-neutralizing agent. N. Engl. J. Med..

[60] Happel K.I., Lockhart E.A., Mason C.M., Porretta E., Keoshkerian E., Odden A.R., Nelson S., Ramsay A.J. (2005). Pulmonary interleukin-23 gene delivery increases local T-cell immunity and controls growth of Mycobacterium tuberculosis in the lungs. Infect. Immun..

[61] Ferreira R.G., Gordon N.F., Stock R., Petrides D. (2021). Adenoviral vector covid-19 vaccines: Process and cost analysis. Processes.

[62] Hu Z., Lu S.H., Lowrie D.B., Fan X.Y. (2022). Research Advances for Virus-vectored Tuberculosis Vaccines and Latest Findings on Tuberculosis Vaccine Development. Front. Immunol..

[63] Santosuosso M., Zhang X., Mccormick S., Wang J., Hitt M., Xing Z. (2005). Protective CD4 and CD8 T Cells within the Airway Lumen 1. J. Immunol..

[64] Xing Z., McFarland C.T., Sallenave J.M., Izzo A., Wang J., McMurray D.N. (2009). Intranasal mucosal boosting with an adenovirus-vectored vaccine markedly enhances the protection of BCG-primed guinea pigs against pulmonary tuberculosis. PLoS ONE.

[65] Pérez De Val B., Villarreal-Ramos B., Nofrarías M., López-Soria S., Romera N., Singh M., Abad F.X., Xing Z., Vordermeier H.M., Domingo M. (2012). Goats primed with Mycobacterium bovis BCG and boosted with a recombinant adenovirus expressing Ag85A show enhanced protection against tuberculosis. Clin. Vaccine Immunol..

[66] Smaill F., Jeyanathan M., Smieja M., Medina M.F., Thanthrige-Don N., Zganiacz A., Yin C., Heriazon A., Damjanovic D., Puri L. (2013). A human type 5 adenovirus-based tuberculosis vaccine induces robust T cell responses in humans despite preexisting anti-adenovirus immunity. Sci. Transl. Med..

[67] Stylianou E., Griffiths K.L., Poyntz H.C., Harrington-Kandt R., Dicks M.D., Stockdale L., Betts G., McShane H. (2015). Improvement of BCG protective efficacy with a novel chimpanzee adenovirus and a modified vaccinia Ankara virus both expressing Ag85A. Vaccine.

[68] Wilkie M., Satti I., Minhinnick A., Harris S., Riste M., Ramon R.L., Sheehan S., Thomas Z.R.M., Wright D., Stockdale L. (2020). A phase I trial evaluating the safety and immunogenicity of a candidate tuberculosis vaccination regimen, ChAdOx1 85A prime—MVA85A boost in healthy UK adults. Vaccine.

[69] Satti I., Meyer J., Harris S.A., Thomas Z.R.M., Griffiths K., Antrobus R.D., Rowland R., Ramon R.L., Smith M., Sheehan S. (2014). Safety and immunogenicity of a candidate tuberculosis vaccine MVA85A delivered by aerosol in BCG-vaccinated healthy adults: A phase 1, double-blind, randomised controlled trial. Lancet Infect. Dis..

[70] Sivakumaran D., Blatner G., Bakken R., Hokey D., Ritz C., Jenum S., Grewal H.M.S. (2021). A 2-Dose AERAS-402 Regimen Boosts CD8+ Polyfunctionality in HIV-Negative, BCG-Vaccinated Recipients. Front. Immunol..

[71] Kagina B.M.N., Tameris M.D., Geldenhuys H., Hatherill M., Abel B., Hussey G.D., Scriba T.J., Mahomed H., Sadoff J.C., Hanekom W.A. (2014). The novel tuberculosis vaccine, AERAS-402, is safe in healthy infants previously vaccinated with BCG, and induces dose-dependent CD4 and CD8T cell responses. Vaccine.

[72] Perera P.Y., Derrick S.C., Kolibab K., Momoi F., Yamamoto M., Morris S.L., Waldmann T.A., Perera L.P. (2009). A multi-valent vaccinia virus-based tuberculosis vaccine molecularly adjuvanted with interleukin-15 induces robust immune responses in mice. Vaccine.

[73] Roediger E.K., Kugathasan K., Zhang X.Z., Lichty B.D., Xing Z. (2008). Heterologous boosting of recombinant adenoviral prime immunization with a novel vesicular stomatitis virus-vectored tuberculosis vaccine. Mol. Ther..

[74] Liu K.Y., Yang W.H., Dong X.K., Cong L.M., Li N., Li Y., Wen Z.B., Yin Z., Lan Z.J., Li W.P. (2016). Inhalation Study of Mycobacteriophage D29 Aerosol for Mice by Endotracheal Route and Nose-Only Exposure. J. Aerosol Med. Pulm. Drug Deliv..

[75] Carrigy N.B., Larsen S.E., Reese V., Pecor T., Harrison M., Kuehl P.J., Hatfull G.F., Sauvageau D., Baldwin S.L., Finlay W.H. (2019). Prophylaxis of mycobacterium tuberculosis H37Rv infection in a preclinical mouse model via inhalation of nebulized bacteriophage D29. Antimicrob. Agents Chemother..

[76] Stukova M. (2018). Randomized Open Label Phase 1 Clinical Trial of TB/FLU-01L Tuberculosis Vaccine Administered Intranasally or Sublingual in BCG-Vaccinated Healthy Adults. Global Forum on TB Vaccines, New Delhi, India. https://tbvaccinesforum.org/wp-content/uploads/2018/03/5GF-Breakout-2-Stukova.pdf.

[77] Shurygina A.P., Zabolotnykh N., Vinogradova T., Khairullin B., Kassenov M., Nurpeisova A., Sarsenbayeva G., Sansyzbay A., Vasilyev K., Buzitskaya J. (2023). Preclinical Evaluation of TB/FLU-04L—An Intranasal Influenza Vector-Based Boost Vaccine against Tuberculosis. Int. J. Mol. Sci..

[78] Hansen S.G., Zak D.E., Xu G., Ford J.C., Marshall E.E., Malouli D., Gilbride R.M., Hughes C.M., Ventura A.B., Ainslie E. (2018). Prevention of tuberculosis in rhesus macaques by a cytomegalovirus-based vaccine. Nat. Med..

[79] Belnoue E., Vogelzang A., Nieuwenhuizen N.E., Krzyzaniak M.A., Darbre S., Kreutzfeldt M., Wagner I., Merkler D., Lambert P.H., Kaufmann S.H.E. (2022). Replication-Deficient Lymphocytic Choriomeningitis Virus-Vectored Vaccine Candidate for the Induction of T Cell Immunity against Mycobacterium tuberculosis. Int. J. Mol. Sci..

